# A Positive Dermcidin Expression Is an Unfavorable Prognostic Marker for Extramammary Paget’s Disease

**DOI:** 10.3390/diagnostics11061086

**Published:** 2021-06-14

**Authors:** Shun Ohmori, Yu Sawada, Natsuko Saito-Sasaki, Sayaka Sato, Yoko Minokawa, Hitomi Sugino, Hikaru Nanamori, Kayo Yamamoto, Etsuko Okada, Motonobu Nakamura

**Affiliations:** Department of Dermatology, University of Occupational and Environmental Health, 1-1, Iseigaoka, Yahatanishi-Ku, Kitakyushu, Fukuoka 807-8555, Japan; oh-sh@med.uoeh-u.ac.jp (S.O.); natsuko-saito@med.uoeh-u.ac.jp (N.S.-S.); a0619gacha@gmail.com (S.S.); min.yo5.0628@gmail.com (Y.M.); hsugino@med.uoeh-u.ac.jp (H.S.); hikaru-n@med.uoeh-u.ac.jp (H.N.); kayohama@med.uoeh-u.ac.jp (K.Y.); e-okada@med.uoeh-u.ac.jp (E.O.); motonaka@med.uoeh-u.ac.jp (M.N.)

**Keywords:** extramammary Paget’s disease, dermcidin, prognosis, lymph node metastasis, survival

## Abstract

Extramammary Paget’s disease is recognized as an apocrine-origin cutaneous tumor and is localized in the intraepithelial skin lesion. However, its advanced form is intractable, and there is currently no therapeutic option with a satisfactory level of clinical outcome. Therefore, it is of great importance to identify a potential biomarker to estimate tumor advancement in extramammary Paget’s disease. Dermcidin is an antimicrobial peptide derived from the eccrine gland and is identified as a biomarker in various malignancies. To investigate the potential of dermcidin in extramammary Paget’s disease, we investigated dermcidin expression in tumors using the immunostaining technique. Although previous studies have reported that extramammary Paget’s disease has no positive staining against dermcidin, 14 out of 60 patients showed positive staining of dermcidin in our study. To clarify the characteristics of positive dermcidin in extramammary Paget’s disease, we investigated the clinical characteristics of positive dermcidin extramammary Paget’s disease patients. Positive dermcidin patients showed a significantly high frequency of lymph node metastasis. We next investigated the impact of positive dermcidin on overall survival. Univariate analysis identified that positive dermcidin showed a significantly increased hazard ratio in overall survival, suggesting that dermcidin might be a prognostic factor for extramammary Paget’s disease.

## 1. Introduction

The skin is a large surface organ in the human body and is complicatedly organized with various unique cells and glands to adjust to external environmental changes [1,2,3]. Due to this unique characteristic of skin as a peripheral organ, various types of tumors, including malignancies, have the chance to emerge in it. In the early phase of tumor development, the tumor is localized in the skin, which can be adequately targeted through skin-focused local treatment, such as surgical resection [4]. However, once it progresses to an advanced form and causes distant organ metastasis, it is intractable due to the limited number of therapeutic options against these metastatic skin cancers [5]. Despite recent advancements in immune checkpoint and molecular targeted therapy, this therapeutic approach has still not reached a satisfactory level to obtain positive clinical outcomes [6].

The origin of extramammary Paget’s disease is believed to be the apocrine glands. This is because the disease usually arises in the genital, perianal, and axillary regions, where the apocrine glands are located [7]. In agreement with this, previous histological studies have identified that extramammary Paget’s disease showed positive immunoreactivity against gross cystic disease fluid protein (GCDFP)-15, carcinoembryonic antigen (CEA), and cytokeratin (CK) 7, all of which receive the same response from the apocrine glands [8]. In general, patients with extramammary Paget’s disease have a good prognosis, with a 5-year overall survival of 75% to 95%, because the disease is localized in the epidermis [4,9,10,11,12]. In contrast, dermal invasion is closely associated with lymph node metastasis and poor prognosis [8]. However, there is a limited number of biomarkers that can help toward the prognosis of extramammary Paget’s disease.

A unique antimicrobial peptide was identified in 2001, i.e., dermcidin., which is constitutively produced by sweat glands. Additionally, an abundance of dermcidin was detected in sweat, showing a beneficial impact on antimicrobial action against microorganisms [13]. In contrast to its beneficial effect on the human body, however, dermcidin can also play an important role in the development of malignant tumors and other diseases. Based on this property of dermcidin, its usefulness as a biomarker for various diseases has been evaluated. Because extramammary Paget’s disease is believed to be a malignant tumor derived from the apocrine glands, representing a negative expression of dermcidin in normal tissue, the possible role of dermcidin as a biomarker in patients with extramammary Paget’s disease has not been investigated.

In this study, we investigated the potential of dermcidin as a biomarker for the prognosis of extramammary Paget’s disease. We identified that there are two groups, dermcidin-positive and -negative extramammary Paget’s disease. In addition, dermcidin-positive patients showed an unfavorable clinical behavior and a high frequency of lymph node metastasis. Our results suggest that dermcidin might be an independent prognostic factor in patients with extramammary Paget’s disease.

## 2. Materials and Methods

### 2.1. Patient Population

In total, 60 patients who underwent surgery as an initial form of treatment for extramammary Paget’s disease at the Department of Dermatology, University of Occupational and Environmental Health, were enrolled in this study from December 1979 to August 2017. The diagnosis was based on histopathological analysis carried out by two independent pathologists. Tissue specimens of the tumor were obtained from patients who underwent surgery at our institution. Because of the rarity of this malignant cutaneous tumor [14], it is sometimes difficult for a diagnosis of extramammary Paget’s disease to be made, especially as it is hard to distinguish the disease from the pagetoid phenomenon. To exclude a pagetoid phenomenon, perianal extramammary Paget’s disease was evaluated via GCDFP15+ and CK20- to determine the correctness of the diagnosis of extramammary Paget’s disease. Patients were categorized according to the degree of dermcidin expression, age, sex, and the presence of depigmentation in the skin lesion.

### 2.2. Immunostaining for Dermcidin

Immunostaining was performed as reported previously [15,16]. In brief, immunochemical staining for dermcidin was conducted using two dermcidin monoclonal antibodies (mAbs) (A-20 and N-20) (Santa Cruz Biotechnology, Santa Cruz, CA, USA) on formalin-fixed, paraffin-embedded specimens. In brief, specimens were cut into 4 μm thick sections and then deparaffinized in xylene and dehydrated through graded alcohol solutions. Antigen retrieval was achieved via boiling in citrate buffer, pH 6.0, using a microwave treatment. All sections were treated with methanol containing 0.3% H_2_O_2_ for 15 min to block endogenous peroxidase activity. Immunoglobulin G was treated using normal rabbit serum (Nichirei, Tokyo, Japan) to avoid nonspecific antibody binding. After overnight incubation at 4 °C with mouse anti-dermcidin mAb (Lifespan BioSciences, Inc., Seattle, Washington, DC, USA), the sections were incubated with biotinylated rabbit–anti-mouse secondary antibody (Nichirei, Tokyo, Japan) followed by incubation in a streptavidin–peroxidase complex solution for 30 min. Signals were generated via incubation with 3-amino-9-ethyl carbazole to visualize the immunostaining. The expression of dermcidin was classified into 2 groups: dermcidin-positive patients and dermcidin-negative patients. Negative dermcidin indicated absolutely no immunostaining reaction to anti-dermcidin antibody in both specific antibodies.

### 2.3. Statistical Analyses

Fisher’s exact test for unpaired data was used to analyze the association between dermcidin expression and various clinicopathologic factors. Univariate analyses of overall survival were conducted using the log-rank test, and Kaplan–Meier curves were generated. Overall survival was calculated from the date of first diagnosis to the date of death or latest contact with the patient. Univariate analysis was performed using the SPSS software (IBM Corp., Armonk, NY, USA). Kaplan–Meier survival analyses and Fisher’s test were performed using GraphPad Prism 4.0. The senser on the survival curve means still alive or discontinuation of follow-up observation during this study period.

### 2.4. Microarray Data Analysis

For microarray data analysis, dermcidin mRNA expression in healthy subject tissues was obtained from a public data set deposited in the National Center for Biotechnology Information (NCBI) obtained from the Gene Expression Omnibus (GEO) database (GEO accession no. GDS3834) [17]. mRNA was extracted from human tissues, which were purchased from commercial vendors and subjected to microarray analysis.

### 2.5. Study Approval

Our retrospective study was approved by the Institutional Review Board at the University of Occupational and Environmental Health following the Declaration of Helsinki. Because this study was a retrospective cohort study, the opt-out method of obtaining informed consent was adopted, and informed consent was waived by the Institutional Review Board at the University of Occupational and Environmental Health.

## 3. Results

### 3.1. The Finding of Dermcidin-Positive Extramammary Paget’s Disease

**Figure 1:**
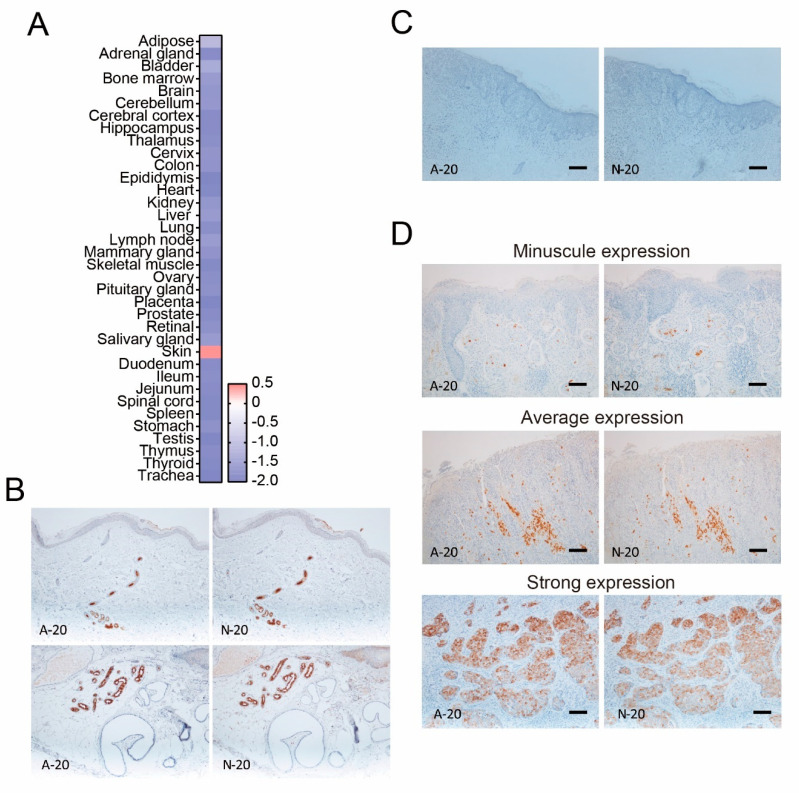
Dermcidin expression in healthy tissue and in the skin and positive staining in extramammary Paget’s disease. (**A**) Microarray dataset analysis of dermcidin gene expression; (**B**) representative dermcidin immunostaining for eccrine glands in healthy subjects using two different immunostaining antibodies; (**C**,**D**) representative negative and (**C**,**D**) positive staining patterns of dermcidin were observed in extramammary Paget’s disease tumor in both intraepithelial and dermal invasive tumors. (**C**) Scale bar: 100 μm; (**D**) scale bar: minuscule expression and average expression were determined at 100 μm, and strong expression was determined at 50 μm.

Dermcidin is produced by the eccrine glands, which are located in the skin. In agreement with this, microarray data set analysis showed that dermcidin expression was highest in the skin from healthy human tissues (Figure 1A). In addition, we confirmed that two antibodies against dermcidin showed a specific positive reaction to the eccrine glands (Figure 1B), suggesting that these antibodies reflect the positivity of dermcidin in the skin.

It has previously been reported that the expression of dermcidin was not identified in epithelial tumors, melanoma, and extramammary Paget’s disease [18]. Although there were cases with no staining of dermcidin (Figure 1C), these antibodies showed that several patients diagnosed with extramammary Paget’s disease had different expression patterns of dermcidin in the tumor, such as minuscule, average, and strong expression (Figure 1D). These unexpected results prompted us to investigate the characteristics of extramammary Paget’s disease with or without dermcidin-positive reaction in further detail.

### 3.2. The Different Characteristics of Extramammary Paget’s Disease Depending on the Expression Degree of Dermcidin

Although extramammary Paget’s disease is usually characterized by no expression of dermcidin, as reported previously, we speculated that there were differences in clinical characteristics in extramammary Paget’s disease between positive and negative expression of dermcidin.

**Figure 2:**
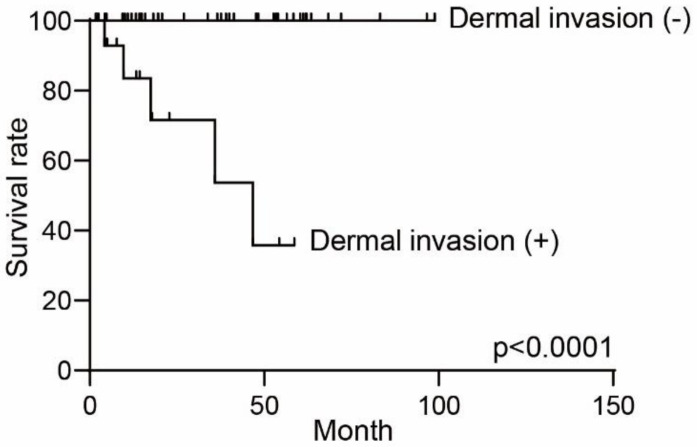
Difference of overall survival curve with or without dermal invasion in extramammary Paget’s disease. The overall survival curves were drawn using the Kaplan–Meier method and were compared with the log-rank test.

To clarify this issue, we investigated the differences in age, sex, depigmentation as the manifestation of extramammary Paget’s disease, and lymph node metastasis between dermcidin high- and low-expressing groups (Table 1 and Table 2). Although there was no significant difference in age, sex, and depigmentation of the tumor, we noticed that the dermcidin-positive group showed a significantly high frequency of nodules and erosion of the tumor upon physical examination. In addition, the dermcidin-positive group also showed a high frequency of dermal invasion and lymph node metastasis. Dermal invasion cases enrolled in this study showed an unfavorable 5-year survival rate of 68.8% (*p* < 0.0001) (Figure 2). These findings suggest that the positive expression of dermcidin might reflect the extension of tumor development.

### 3.3. The Different Prognosis in Extramammary Paget’s Disease

**Figure 3:**
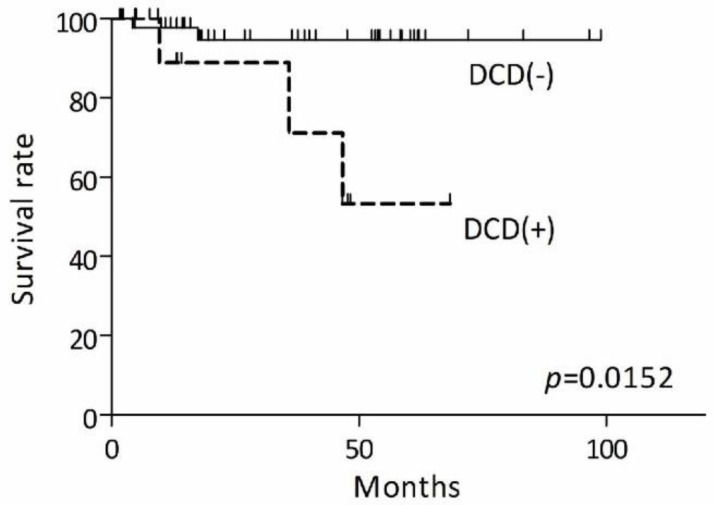
Differences in overall survival curve with or without dermcidin expression in extramammary Paget’s disease. The overall survival curves were drawn using the Kaplan–Meier method and were compared with the log-rank test.

We next investigated the prognostic impact of dermcidin in extramammary Paget’s disease. The mean survival times were different between positive and negative dermcidin expression in the tumor. Kaplan–Meier curves of overall survival are shown in Figure 3. The overall survival rate in dermcidin-positive patients was significantly lower than that in dermcidin-negative patients. Therefore, a high expression of dermcidin is associated with the poorest prognosis.

Finally, we conducted univariate analyses of dermcidin expression in comparison with clinical variables (Table 3). Univariate analysis showed significantly increased hazard ratios in nodules upon physical examination and dermal invasion and lymph node metastasis in the histological examination, consistent with previous studies [4,19]. In addition, a high expression of dermcidin leads to a significantly increased hazard ratio. Although the impact of dermcidin on prognosis might be limited, dermcidin might become a tool for estimating prognosis in patients with extramammary Paget’s disease in some cases.

## 4. Discussion

This study revealed that positive dermcidin expression reflects unfavorable clinical behavior in extramammary Paget’s cell tumors. Cancer cell migration into lymph nodes is an important step in the progression toward the advanced stage of malignant tumors. However, the detailed molecular mechanism determining whether dermcidin promotes such tumor cell migration remains unclear.

Our study showed that dermcidin-positive extramammary Page’s disease exhibited a high frequency of nodules upon physical examination. One of the reasons behind this might be that dermcidin contributes to the development of the tumor. High dermcidin expression is associated with tumor growth in gastric [20] and breast cancer [21]. Interestingly, dermcidin is also associated with tumor growth and tumor apoptosis in breast cancer. As regards the mechanisms, dermcidin has been found to modulate the HER-2-mediated signal pathway [21], which is one of the major pathways in breast cancer [22]. Because HER-2 signaling is also involved in the pathogenesis of extramammary Paget’s disease [23], it is assumed that dermcidin might also activate HER-2 signaling in extramammary Paget’s disease and subsequently lead to the development of tumor growth. Because there was no commercially available cell line of extramammary Paget’s disease, however, further investigation will be required to clarify the detailed molecular mechanisms.

Several studies have already shown the potential of dermcidin as a biomarker for malignancies. A high expression of dermcidin was identified in approximately 10% of breast cancer patients and has been found to be closely associated with the advanced clinical stage and unfavorable clinical behavior due to regulation of tumor cell growth [24]. Serum dermcidin levels were significantly increased in hepatocellular carcinoma patients and were positively correlated with metastasis [25]. Dermcidin expression in gastric cancer reflects overall survival and is positively correlated with lymph node metastasis [20]. Dermcidin expression is higher in lung cancer patients compared with that in healthy subjects [26]. Among cutaneous malignancies, having high serum levels of dermcidin at the moment of melanoma diagnosis has been associated with the metastatic progression of melanoma among melanoma patients [27,28].

Dermcidin has also been reported as a biomarker in various diseases in addition to malignant tumors. Dermcidin has been identified as a biomarker for Alzheimer’s disease (AD) [29], asthma [30], acne vulgaris [31,32], severe obstructive sleep apnea [33], and facioscapulohumeral muscular dystrophy [34]. For example, an abundance of dermcidin was identified in exhaled breath condensate in asthma patients [30]. Therefore, dermcidin may also be a potential biomarker in a variety of skin diseases.

The reason that positive dermcidin expression was observed in clinical patients with unfavorable outcomes who suffered from extramammary Paget’s disease remains unclear. A previous study suggested the possibility that one of the characteristics of the eccrine glands might be linked to extramammary Paget’s disease. The expressions of histoblood group A type 1, 2, and 3 antigens in normal human skin and extramammary Paget’s disease were examined via the immunohistochemical technique [35]. The eccrine glands expressed these antigens, while a negative expression of these antigens was observed in apocrine glands, suggesting that extramammary Paget’s disease might be an apocrine-gland-derived tumor stemming from a negative reaction to these antigens. However, 7 out of 16 cases were positive for these antigens, and 6 out of 7 positive cases were associated with dermal invasion. Meanwhile, 5 cases without dermal invasion were negative against these antigens. Although there has been a limited number of studies focusing on this issue to date, the possibility still exists that eccrine gland characteristics include the development of extramammary Paget’s disease.

A previous study showed a negative dermcidin expression in patients with extramammary Paget’s disease [18]. However, we speculated that the reason behind this result might be that this study did not include any unfavorable clinical cases to show a representative tumor phenotype of extramammary Paget’s disease, which is generally located in the epidermis. This previous study may have selected noninvasive extramammary Paget’s disease samples to visualize a representative sample of an indolent cutaneous tumor from an extramammary Paget’s disease patient.

One possible limitation of our study was that the number of patients involved might not be sufficient to investigate the more detailed characteristics of dermcidin-positive patients with extramammary Paget’s disease. Additionally, the detailed molecular role of dermcidin in extramammary Paget’s disease for the invasion and metastasis of tumors still needs to be clarified, especially how the degree of dermcidin is associated with the activation of metastatic factors and tumor development, which are involved in the molecular mechanism mediated by HER-2 signaling.

The reason that many of the censored cases are in the dermcidin-negative survival curve may be related to the characteristics of indolent-type cutaneous malignancy. Patients with the nondermal invasion type had their clinical observation follow-up in other hospitals after surgical resection in our department. By contrast, dermal invasion cases are known to have an unfavorable clinical behavior, as shown in Figure 2, and thus, careful follow-up was needed in our hospital or in another hospital where skin oncologists are available.

In conclusion, dermcidin has the potential to help toward the prognosis of extramammary Paget’s disease at the moment of surgical resection of the tumor. It is therefore urgently needed to further investigate the actual impact of dermcidin on the molecular mechanism of the development of extramammary Paget’s disease.

## Figures and Tables

**Table 1:** Clinical characteristics of extramammary Paget’s disease patients in this study.

Variable		Patients Number
Total		60
Age		
	<60	3
	60–69	13
	70–79	28
	80–89	13
	≥90	3
Sex		
	Male	34
	Female	26
Primary site		
	Genital	54
	Genital and axillary	1
	Genital, axillary, and navel	1
	Perianal	2
	Axillary	1
	Back	1
Clinical manifestations		
	Nodule	9
	Erosion	33
	Depigmentation	21
Dermal invasion		
	Absent	44
	Present	16
Lymph node metastases		
	Absent	52
	Present	8

**Table 2:** Difference in clinical characteristics in dermcidin expression.

Variable		Total	Dermcidin (+)	Dermcidin (-)	*p* Value
Total		60	14	46	
Age					0.314
	<70	16	2	14	
	≥70	44	12	32	
Sex					1.000
	Male	34	8	26	
	Female	26	6	20	
Nodule					0.003
	Absent	51	8	43	
	Present	9	6	3	
Erosion					0.013
	Absent	27	2	25	
	Present	33	12	21	
Depigmentation					1.000
	Absent	39	9	30	
	Present	21	5	16	
Dermal invasion					0.006
	Absent	44	6	38	
	Present	16	8	8	
Lymph node metastases					0.013
	Absent	52	9	43	
	Present	8	5	3	

**Table 3:** Univariate analysis of clinical variables.

Variable		HR	95% CI	*p* Value
Age				0.234
	<70	1		
	≥70	0.3262	0.05144–2.068	
Sex				0.709
	Male	1		
	Female	1.398	0.2406–8.117	
Nodule				<0.0001
	Absent	1		
	Present	7561	338.7–168,800	
Erosion				0.275
	Absent	1		
	Present	2.671	0.4570–15.60	
Depigmentation				0.432
	Absent	1		
	Present	0.4823	0.07834–2.969	
Dermal invasion				<0.0001
	Absent	1		
	Present	240.8	24.80–2338	
Lymph node metastases				<0.0001
	Absent	1		
	Present	442,600	16,480–11,880,000	
dermcidin expression				0.015
	Absent	1		
	Present	16.79	1.721–163.7	

## Data Availability

Not applicable.
